# Neovascular age-related macular degeneration complicated by large submacular hemorrhage managed with faricimab monotherapy

**DOI:** 10.1186/s40942-025-00771-5

**Published:** 2025-11-22

**Authors:** Jerald Lim, Hannah Cho, Hema Ramkumar, Sean D. Adrean

**Affiliations:** 1 University of California, Irvine School of Medicine, Irvine, CA 92617 USA; 2 Retina Consultants of Orange County, 301 W. Bastanchury Road #285, Fullerton, CA 92835 USA

**Keywords:** Faricimab, Neovascular age-related macular degeneration, Subretinal hemorrhage

## Abstract

**Purpose:**

To retrospectively evaluate the visual and anatomic outcomes of intravitreal faricimab monotherapy in treatment naïve neovascular age-related macular degeneration (nAMD) patients presenting with large submacular hemorrhage (SMH).

**Methods:**

Patients with neovascular age-related macular degeneration (nAMD) presenting with at least 50% subretinal hemorrhage (SRH) involving the fovea between March 2023 and June 2025 were identified. Patients with greater than 750 microns of total thickness of subfoveal hemorrhage were excluded. Patients were treated with faricimab as first line therapy utilizing a treat-extend-stop protocol for one year. Spectral domain optical coherence tomography (SD-OCT) and fluorescein angiogram (FA)/ indocyanine green angiography (ICG) were used to determine total CNV lesion size, SRH area size, and macular thickness. Visual acuity and SD-OCT were also obtained at each visit.

**Results:**

Six patients were identified that met the inclusion criteria. There were two males and four females. The average age of was 83.0 years. Prior to treatment, the average total CNV lesion size was 19.7mm^2^ and the average hemorrhage area was 14.5 mm^2^ for an average SRH percentage of 73.8%. The average central macular thickness (CMT) prior to treatment was 478.3 μm and the average CMT 6 months after beginning treatment was 306.8 μm (*p* = 0.0031). The average CMT 1 year after treatment was 259.0 μm (*p* = 0.0017). Average ETDRS letters before treatment was 48.8 letters {Snellen equivalent 20/138} with a range of 23–70 letters. Average ETDRS letters after 6 months of treatment was 60.3 letters {Snellen equivalent 20/63} with a range of 42 to 76 letters (*p* = 0.017). Average ETDRS letters after 1 year of treatment was 64.7 letters {Snellen equivalent 20/50} with a range from 26 to 80 letters (*p* < 0.014). The average number of injections was 9.3 at 1 year.

**Conclusion:**

In patients with nAMD complicated by at least 50% SRH, there was improved vision and decreased CMT. On average, vision improved by 16.4 ETDRS letters and improved on average from 20/138 to 20/50 on Snellen after 1 year of faricimab monotherapy. These patients may be successfully managed with faricimab monotherapy. While visual acuity overall improved, some patients had more improvement than others likely due to the location of the CNV.

## Introduction

In January 2022, faricimab (Vabysmo, San Francisco, CA, Genentech) was approved in the United States as a treatment for patients with neovascular (wet) age-related macular degeneration (nAMD) and diabetic macular edema (DME) [1]. Administered by intravitreal injection, faricimab is a humanized bispecific IgG1 monoclonal antibody that inhibits both vascular endothelial growth factor (Anti-VEGF) and angiopoietin-2 (Ang-2). Ang-2 expression is increased in inflammation and many different types of cancer, and is typically associated with angiogenesis and pathological vascular reconstruction [2]. Thus, many drugs and therapeutics look to target this protein and block its interaction with the tyrosine kinase with immunoglobulin and epidermal growth factor homology domain 2 (Tie2) receptor. Blocking this pathway has been shown to improve the course of many diseases by reducing inflammation and the angiogenic effects of the Ang2-Tie2 pathway [2]. Similarly, an increase in VEGF expression is also associated with angiogenic growth; it is commonly implicated in many retinal diseases such as diabetic retinopathy and nAMD [3]. Treatments dedicated to blocking this pathway have also been shown to be effective in minimizing disease progression and retinal damage [3]. Faricimab simultaneously inhibits both of these pathways, and it has been shown to inflammation, subretinal macrophage infiltration, and shows promise in reducing fibrosis progression [4, 5]. As a result, faricimab has become a common treatment for nAMD patients.

For patients with nAMD, the current standard of care consists of intravitreal injections including ranibizumab, off-label bevacizumab, aflibercept both 2 and 8 mg, and faricimab. Patients with nAMD may present with or develop a subretinal hemorrhage, which typically leads to photoreceptor damage and eventual vision loss [6]. In the phase 3 Tenaya/Lucerne trial for faricimab, patients were excluded if SRH was >50% of the total lesion area. These patients may still be treated with anti-VEGF therapy, but further studies are necessary to determine the effects of faricimab on this population of patients. In this study, a cohort of treatment naïve patients with nAMD presenting with >50% SRH that involved the macula, were treated with faricimab monotherapy, utilizing a treat-extend-stop protocol [7]. Its efficacy in improving the subretinal hemorrhage and visual acuity was recorded.

## Materials and methods

Local institutional review board (IRB) approval was obtained, IRB00012874; 2025-002-RCOC. All data was collected in accordance with the Health Insurance Portability and Accountability Act (HIPAA). This study adhered to the tenets of the Declaration of Helsinski.

This retrospective study identified treatment naïve patients between March 2023 and June 2025 with nAMD that presented with subretinal hemorrhage that was greater than 50% of the total CNV lesion size and involved the fovea. Patients with greater than 750 microns of submacular hemorrhage (SMH) were excluded from the analysis, as these patients in our practice receive a pneumatic retinopexy, vitrectomy surgery, or a combination of both. Patients that did not receive one year of faricimab therapy were also excluded from the analysis. Previously treated patients with nAMD that developed a submacular hemorrhage were also excluded. Visual acuity and CMT on SD-OCT were obtained at every visit. At the initial visit, fundus photos (FP), FA and ICG angiography were performed. The lesion size was measured and recorded at the initial visit and at the one-year time point. Time interval between treatments and total number of treatments were obtained and analyzed.

**Fig. 1:**
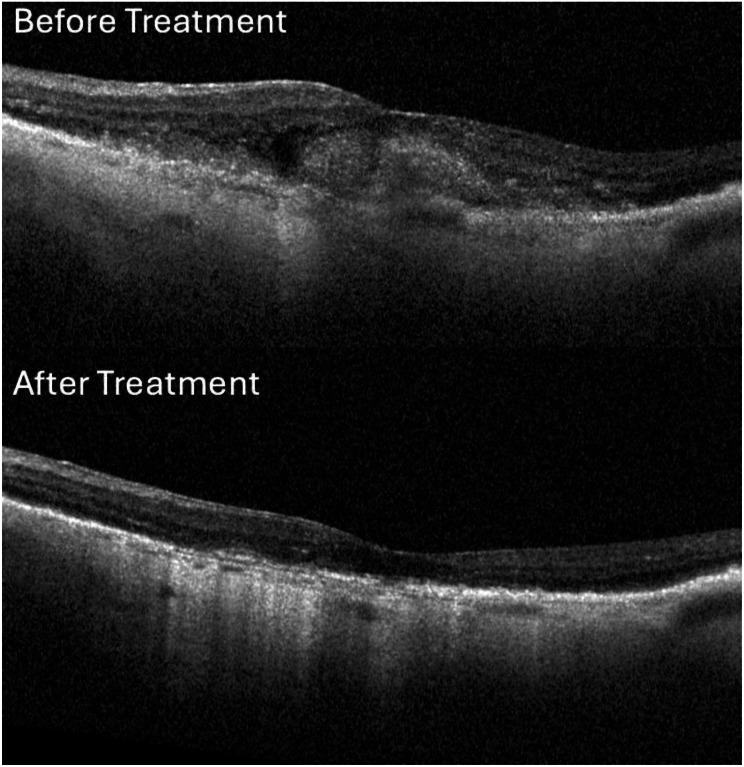
Optical coherence tomography of submacular hemorrhage. OCT of SMH shown before and after 1 year of treatment with faricimab monotherapy showing resolution of hemorrhage

**Fig. 2:**
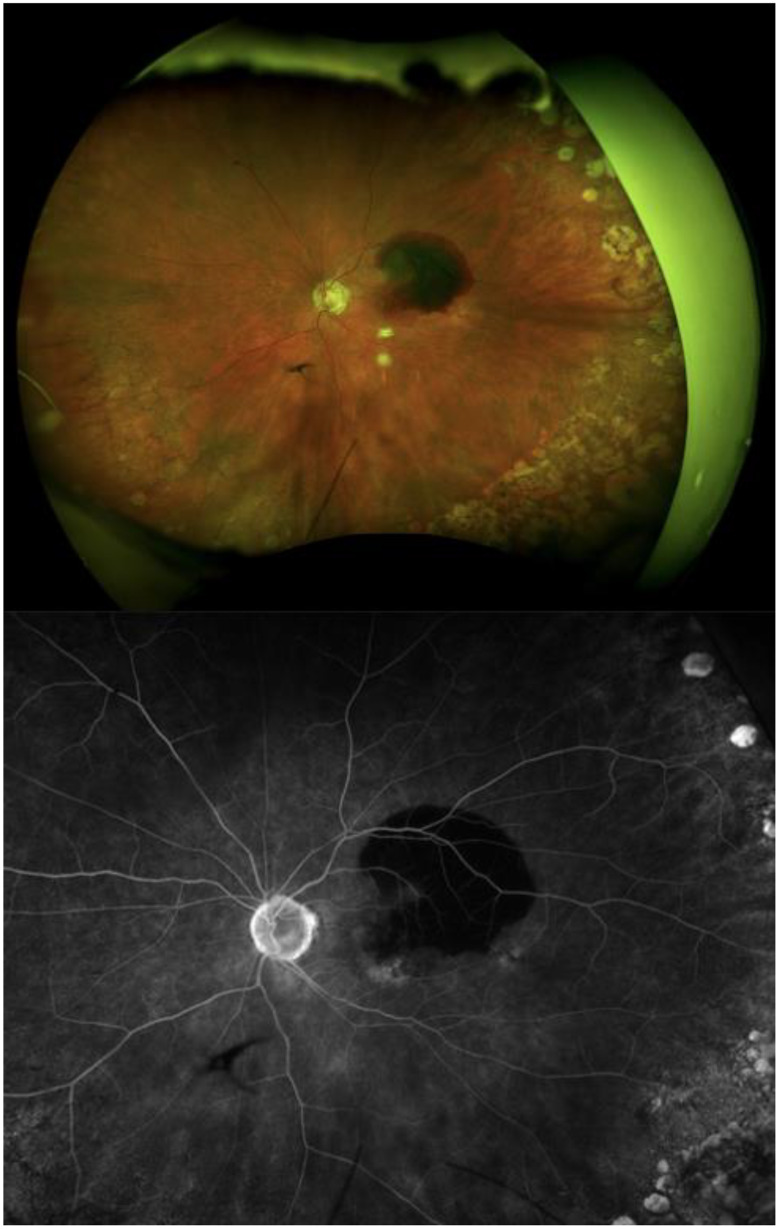
Optos fundus imaging and fluorescein angiogram (FA). Fundus image and FA of patient #6 with submacular hemorrhage (SMH) shown in the image above

An informed consent process was performed for the six treatment naïve patients identified with nAMD complicated by SMH. These patients were treated with faricimab according to the previously described treat-extend-stop (TES) protocol [7], where patients receive at least 3 monthly injections of faricimab until the macula was free of fluid, confirmed by SD-OCT (Fig. 1). Intervals between injections could then be extended by 1–2 weeks between visits until a time interval of 12 weeks was reached, should the macula remain dry [7]. Fundus photos, FA with SD-OCT were used to calculate the subretinal hemorrhage area and total lesion size at presentation (Fig. 2). Subretinal hemorrhage area was then divided by total lesion size to obtain the percentage of the CNV lesion complicated by SRH. Visual acuity (VA) was also measured and converted to Early Treatment Diabetic Retinopathy Study (ETDRS) letters for analysis.

## Results

**Fig. 3:**
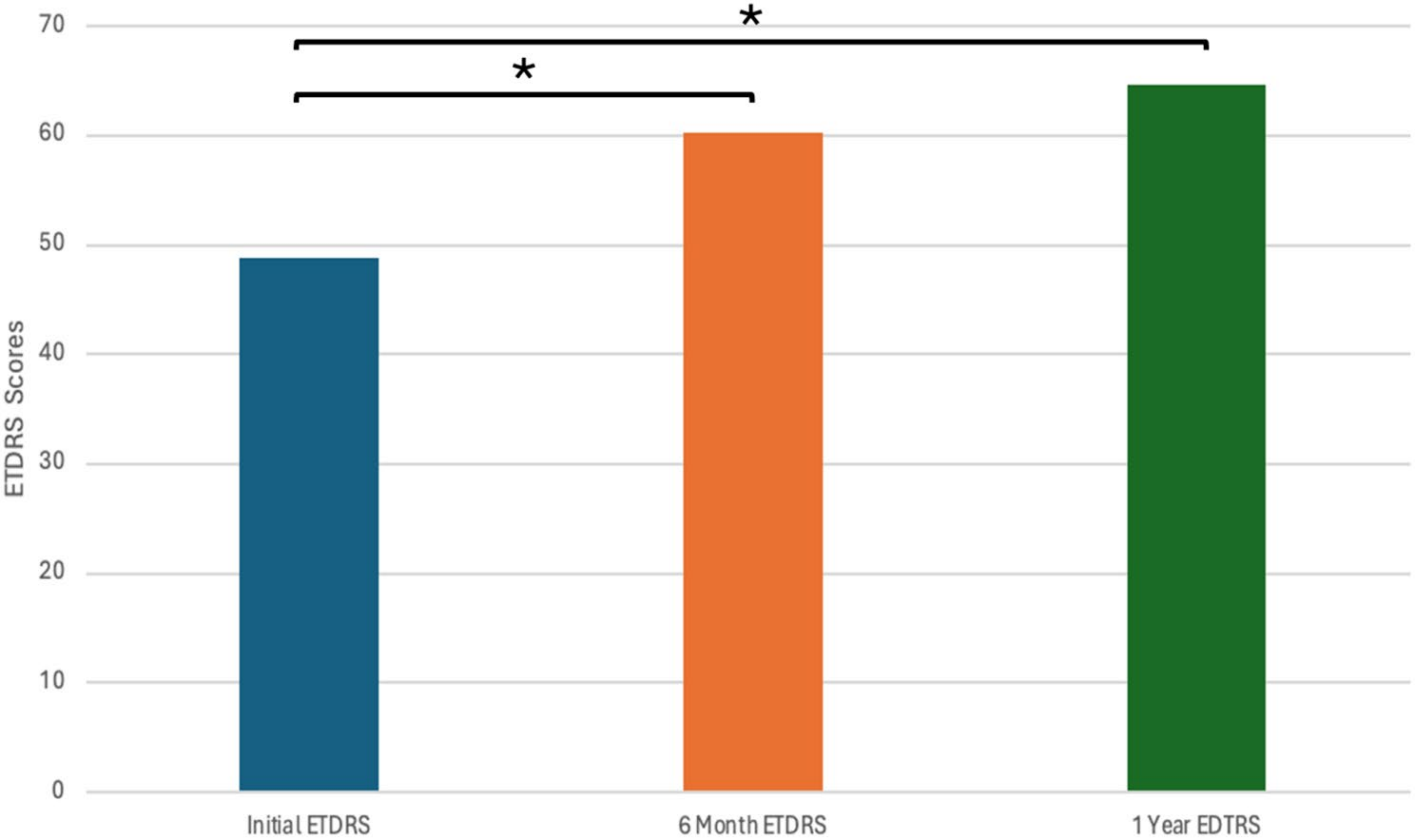
Patient distribution of Snellen visual acuity before, after 6 months, and after 1 year of Faricimab monotherapy. Number of patients in three visual acuity categories (20/20–20/40, 20/50 − 20/150, 20/200 and worse) are recorded and compared before treatment, after 6 months of treatment, and after 1 year of treatment

**Fig. 4:**
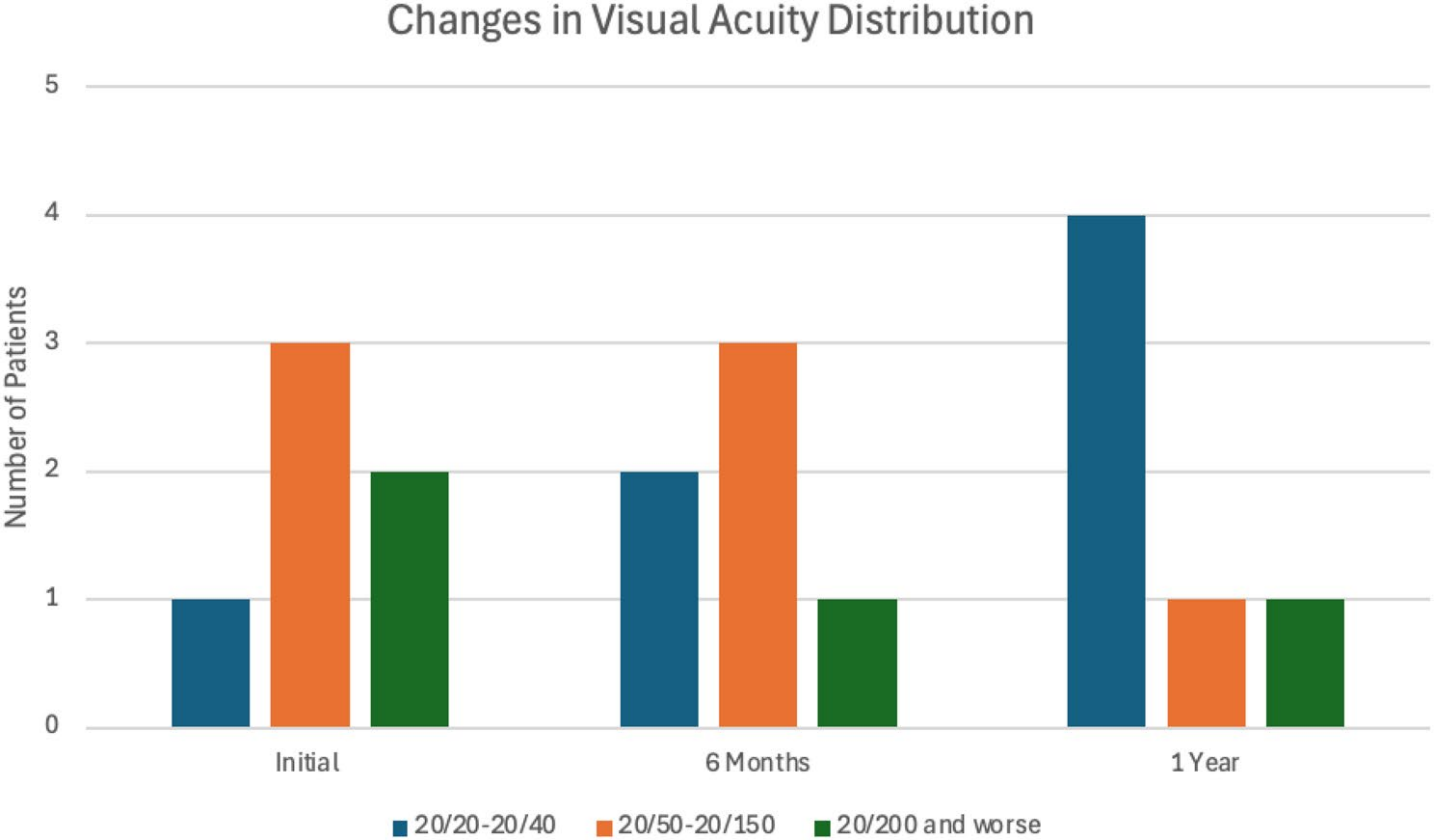
ETDRS scores before treatment, after 6 months, and after 1 year of faricimab monotherapy. Average initial ETDRS is 48.83. Average ETDRS after 6 months is 60.33 (t-test; *p* = 0.017). Average ETDRS after 1 year is 64.67 (t-test; *p* = 0.014)

**Fig. 5:**
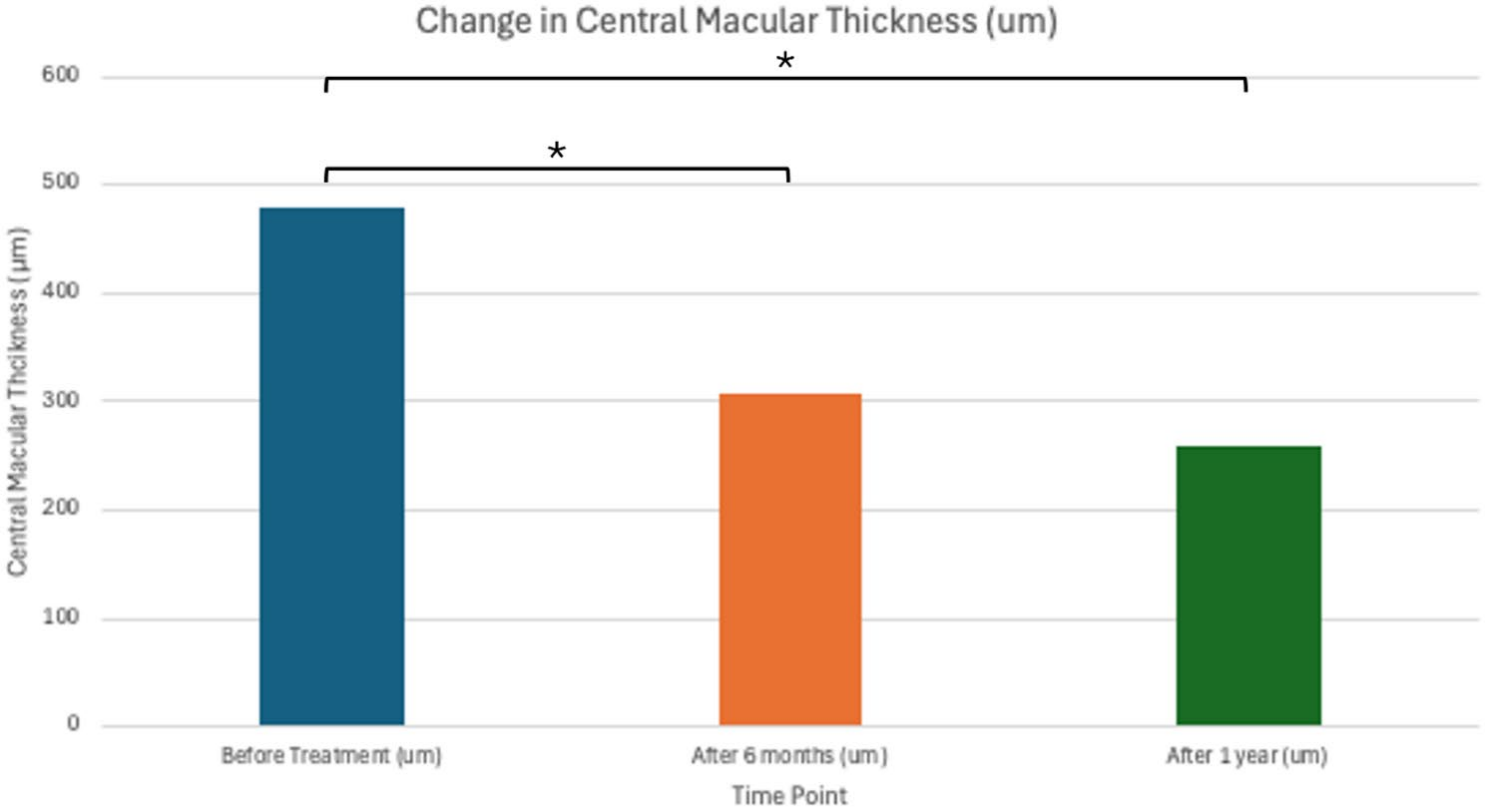
Average central macular thickness before treatment, after 6 months, and after 1 year of treatment with faricimab monotherapy. Average CMT before treatment is 478.3 μm. Average CMT after 6 months is 306.8 μm (t-test; *p* = 0.0031). Average CMT after 1 year is 259.0 μm (t-test; *p* = 0.0017)

A total of 317 treatment naïve patients with nAMD were identified during the study period. Ten patients were identified that had a subretinal hemorrhage from CNV involving the fovea. One patient was excluded since they had started with faricimab, but were then switched to bevacizumab after two injections due to insurance. Three patients were excluded since they were previously treated with anti-VEGF therapy. A total of six patients (1.9% of the treatment naïve study population) met inclusion criteria. There were two males and four females. The mean age was 83.0 years. Prior to treatment, the mean SRH area was 14.5 mm^2^ and the mean total CNV lesion size was 19.7 mm^2^ for an average SRH percentage of 73.8%. At treatment onset, the mean ETDRS vision was 48.3 letters (Snellen equivalent: 20/138) with a range of 23–70 letters. After 6 months of treatment, the average ETDRS letters was 60.3 letters (Snellen equivalent: 20/63) with a range of 30–76 letters (*p* = 0.017). After 1 year of treatment, the average ETDRS letters was 64.7 (Snellen equivalent: 20/50) with a range of 26–80 (*p* = 0.014). (Figs. 3 and 4; Table 1). Average CMT prior to treatment was 478.3 μm, average CMT was 306.8 after 6 months (*p* = 0.0031), and average CMT after 1 year of treatment was 259 μm (*p* = 0.0017) (Fig. 5; Table 1).

**Table 1:**
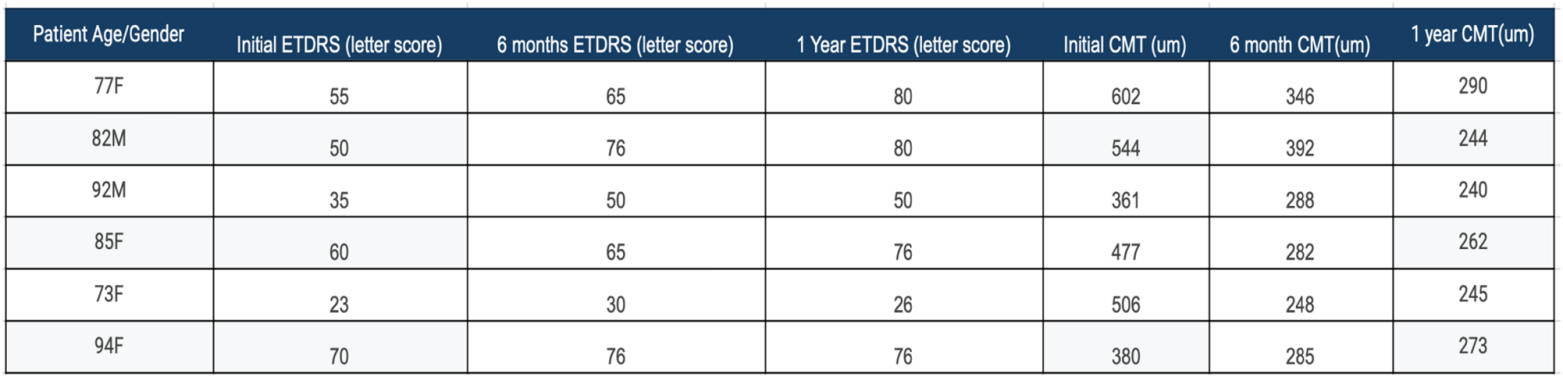
Patient data Table. Patient age and gender depicted. ETDRS letter scores and CMT (um) shown for each patient prior to treatment, 6 months after treatment, and 1 year after treatment with faricimab monotherapy

This cohort of patients were only treated with faricimab monotherapy. No other treatments were required such as intravitreal gas or surgery. The average number of injections was 5.7 after 6 months and 9.3 after 1 year. The patients at the end of year one had a typical injection frequency of every 6.5 weeks. On average, patients saw resolution of subretinal hemorrhage with 2.7 injections. Five patients had resolution with 3 injections, while one patient had resolution with 1 injection of faricimab. At 1 year, 3 patients had subretinal fibrosis. One patient had fibrosis in the fovea while 2 patients had extrafoveal subretinal fibrosis. No patients developed geographic atrophy during the study period. There were typical side effects of injections reported such as discomfort after the injection and occasional subconjunctival hemorrhages. There were no incidents of endophthalmitis, idiopathic occlusive vasculitis or inflammation.

## Discussion

Patients with nAMD complicated by SRH may have severe vision loss [8]. One of the end-stage complications of SRH includes subretinal fibrosis, which is characterized by the formation of fibrous tissue within the subretinal and subretinal pigment epithelial (RPE) space as a result of the CNV. The extent and location of fibrosis can cause damage to various structures including photoreceptors, retinal pigment epithelium, and vessels, ultimately leading to permanent vision loss [9]. Additionally, SRH can contribute to a toxic effect due to iron released from erythrocytes [6]. The iron can be subsequently phagocytosed by macrophages and may cause oxidative stress and damage to the choriocapillaris and photoreceptors. These complications point to the need for prompt and effective treatment to preserve structural function and viability. Intravitreal injections, such as faricimab, are one such way to manage this condition.

In the phase 3 Tenaya/Lucerne clinical trials for faricimab, patients with greater than 50% SRH were excluded from studies [10]. This is typical for many randomized clinical trials for nAMD. However, patients with greater than 50% SRH can be managed similarly to those with less than 50% SRH and see similar improvements in VA, as demonstrated in the study Comparison of Age-Related Macular Degeneration Treatment Trials (CATT) [11]. Those patients were randomized to 4 different groups based on anti-VEGF injections (ranibizumab or bevacizumab) and treatment interval time (monthly vs. PRN). At baseline, they found that patients with greater than 50% SRH had worse visual acuity than those with less than 50% (56.0 to 60.9 letters, *p* = 0.002). Despite this, both groups showed a parallel pattern of visual improvement over time. Specifically, patients with greater than 50% SRH gained an average of 9.3 letters at 1 year and 9 letters at 2 years, similar to those with less than 50% SRH (7.2 and 6.1 letters, respectively). This improvement was accompanied by resolution of hemorrhage and reduction in lesion size [11]. Another retrospective study done by Dimopoulos et al. examining off-label intravitreal bevacizumab therapy found a faster resolution of submacular hemorrhage with moderate visual improvement and with almost complete reabsorption of hemorrhage at 1 year [12]. In this study, the average resolution of subretinal hemorrhage was observed after 2.7 injections. In the Dimopoulos et al. study, the mean best corrected visual acuity (BCVA) improved from 0.81 logMAR (approximately 20/129) at baseline to 0.75 logMAR (approximately 20/112) at 12 months, with over half of the patients gaining one or more ETDRS lines of vision. These two studies show that patients with extensive hemorrhagic involvement can be managed similarly to those with less hemorrhage, with comparable improvements in both visual acuity and anatomic outcomes. This study found that in patients presenting with large submacular hemorrhage treated with faricimab monotherapy had greater and more rapid improvement in patient’s visual acuity and reduction in hemorrhage size compared to the previous studies. In our study, the average visual acuity in our study improved from 48.8 to 60.4 ETDRS letters {Snellen equivalent: 20/138 to 20/63} in 6 months and further improved to 64.7 ETDRS letters {Snellen equivalent: 20/50} after 1 year of treatment following TES protocol.

In addition, there was also significant reduction in CMT with an improvement from 478.3 μm to 306.8 μm after 6 months and further improvement to 259.0 μm after 1 year of treatment following the TES protocol. Therefore, faricimab monotherapy may lead to more rapid and substantial improvements in visual acuity and reduction of SRH and CNV size compared to other VEGF inhibitors, possibly due to its dual Tie-2 and VEGF pathway inhibition.

In this study, we limited the amount of SRH and sub RPE hemorrhage to less than or equal to 750 microns. Aside from anti-VEGF injections, several therapeutic options for SRH include vitreoretinal surgery and pneumatic displacement (PD) that may include adjunctive injection of tissue plasminogen activator (tPA), a protease that cleaves plasminogen into plasmin to degrade and dissolve the fibrin clots [13]. It is the protocol of this practice where this study was performed, to intervene more aggressively when the SRH is greater than 750 microns, utilizing both anti-VEGF therapy and either a PD or vitreoretinal surgery.

Pneumatic displacement (PD) utilizes intravitreal injection of inert gases such as sulfur hexafluoride (SF_6_) or octafluoropropane (C_3_F_8_) to displace blood away from the fovea [14]. This procedure can be preceded or followed with injected tissue-type plasminogen activator (tPA) and is usually used in conjunction with intravitreal anti-VEGF therapy. One retrospective study that assessed the outcomes of treating submacular hemorrhage with anti-VEGF therapy and PD showed improved visual outcomes and successful displacement in 91.7%, with 44% achieving complete resolution and 56% showing inferior displacement with foveal clearing [15]. Adjuvant anti-vascular endothelial growth factor (anti-VEGF) therapy, administered in 75% of cases, played a crucial role in preventing recurrence and managing neovascularization in CNV-related SMH.

Alternatively, vitrectomy is a more invasive and aggressive treatment. One approach involves creating a retinotomy to extract the clot and underlying neovascularization [16]. This also may be performed if a PD fails. A variation of this method includes injecting tPA. Administering tPA may reduce the damage to the outer retina and retinal pigment epithelium. While it is typically injected into the subretinal space, it can also be injected 24 h prior to vitrectomy in patients with substantial submacular hemorrhages. Although vitrectomy is considered effective in achieving short-term improvements in visual acuity for SRH, one study found that at eight months postoperatively, visual acuity declined due to recurrent neovascularization, macular atrophy and fibrosis from neovascular AMD in a majority of the patients [13]. The procedure achieved high anatomical success with effective subretinal displacement of blood using tPA, and no intraoperative complications related to tPA injection were reported. However, postoperative complications, such as retinal detachment (6.6%), vitreous hemorrhage (7.7%), and recurrent SMH (11%), highlight risks that may temper the overall efficacy [17]. Despite these complications, the study found that the surgery was effective in temporarily restoring vision, emphasizing its value as an intervention for larger and thicker submacular hemorrhage management [17]. While both PD and vitrectomy are established treatment options for subretinal hemorrhage (SRH), faricimab monotherapy emerges as an important addition to these existing options, offering a less invasive approach while also providing promising outcomes. This is especially true in patients with subretinal hemorrhages that are less than 750 microns, such as was examined in this study. Surgical intervention is typically reserved for patients with very thick and large subretinal and sub-RPE hemorrhages.

Comparing these therapies highlight the value of anti-VEGF therapy as a safe and effective treatment for SRH, useful as a complement and standalone in terms of safety and outcomes. Taken together, the recent advancements and promise shown in anti-VEGF therapies, such as faricimab, underscore its potential as a first-line agent in this difficult to treat subset of nAMD patients.

In our small cohort of patients, the only complications seen were typical of intravitreal injections such as discomfort at the injection site and subconjunctival hemorrhage. Our study is limited by a small number of patients in the cohort and the retrospective nature of the study.

Further areas of study should include randomized clinical trials that enroll a larger cohort of patients with nAMD complicated by a large SRH with subfoveal hemorrhage and compare faricimab monotherapy to other anti-VEGF therapies. This could be performed in both treatment naïve and treatment experienced patients since this is a rare initial presentation of nAMD, only 1.9% of our treatment naïve study population, making it a difficult subset of patients to identify.

## Conclusion

In this study, patients with nAMD complicated by > 50% SMH saw significant improvements in visual acuity and central macular thickness with intravitreal faricimab injections. Vision improved, on average, by 16.4 letters and patients improved from on average of 20/138 to 20/50 after 1 year of faricimab monotherapy. Thus, faricimab is an effective choice to treat nAMD patients even in cases complicated with > 50% SMH with less than 750 microns of thickness of subfoveal hemorrhage.

## Data Availability

No datasets were generated or analysed during the current study.

## Declarations

### Meeting presentation

American Society of Retina Specialists annual meeting, Long Beach, CA 7/31/2025.
